# Radar-Based Heart Rate Estimation Method Under Respiratory Harmonic Interference

**DOI:** 10.3390/s26175587

**Published:** 2026-09-03

**Authors:** Didi Xu, Ying Li, Zinan Wu, Tingting Xie, Pengwei Gong

**Affiliations:** Beijing Institute of Radio Metrology and Measurement, Beijing 100854, China; xudd201907@163.com (D.X.); liying_bjut@163.com (Y.L.); wzn_uestc@163.com (Z.W.); tingtingx96@163.com (T.X.)

**Keywords:** non-contact vital sign monitoring, complementary ensemble empirical mode decomposition (CEEMD), harmonic signals, frequency-modulated continuous-wave (FMCW), heart rate

## Abstract

Non-contact vital sign monitoring using millimeter-wave radar has emerged as a promising alternative to contact-based devices for continuous healthcare and elderly care applications. However, accurate heart rate estimation remains challenging because the weak cardiac-induced chest displacement is approximately an order of magnitude smaller than respiratory motion, and its fundamental frequency is frequently masked by higher-order respiratory harmonics. Here we propose a signal processing framework that addresses this challenge through three integrated stages: a slow-time phase correlation method that enhances the signal-to-noise ratio by coherently aggregating vital sign energy from adjacent range bins; an adaptive harmonic matching filtering approach based on complementary ensemble empirical mode decomposition that isolates and suppresses respiratory harmonic interference; and autocorrelation-based heart rate estimation. Experimental results obtained with a 77 GHz FMCW radar demonstrate that the proposed method achieves heart rate estimates within 5% error of reference wearable sensors in the presence of respiratory harmonics, with robustness confirmed through long-duration testing. This framework provides a practical solution for reliable radar-based heart rate monitoring without requiring subject-specific calibration or specialized hardware modifications.

## 1. Introduction

Non-contact vital sign monitoring using radar has attracted growing attention as a solution for continuous health assessment in clinical and home environments [1,2,3]. Unlike contact-based electrocardiography and photoplethysmography sensors, which can cause discomfort during prolonged wear and restrict user mobility, radar-based systems enable the unobtrusive measurement of respiration and heart rate without physical contact or privacy concerns [4,5,6]. Among various radar architectures, frequency-modulated continuous-wave millimeter-wave radar is particularly well-suited for vital sign measurement due to its high range resolution, phase sensitivity to sub-millimeter displacements, and ability to operate across diverse environments, including in-vehicle cabins and intensive care settings [7,8,9]. Recent surveys have systematically documented the rapid progress in radar-based non-contact sensing, highlighting its potential for the early diagnosis of cardiovascular and respiratory conditions [2,10].

Radar-based vital sign monitoring relies on phase analysis of reflected signals to reconstruct chest-wall displacements induced by cardiopulmonary activity. A fundamental challenge arises from the disparity in displacement magnitude: cardiac-induced thoracic motion is approximately 0.1–0.5 mm, roughly one order of magnitude smaller than the respiratory motion of 1–12 mm [11,12]. The reconstructed displacement signal therefore contains not only the fundamental respiration and heartbeat frequencies but also multiple respiratory harmonics that extend into the cardiac frequency band [13,14,15]. As a result, the weak heartbeat component is frequently obscured by these harmonic interferences, making accurate heart rate estimation difficult, particularly when a respiratory harmonic coincides with or lies close to the cardiac fundamental frequency [12,16]. This interference problem is widely recognized as a primary obstacle to reliable radar-based heart rate monitoring [17,18,19].

Various approaches have been proposed to mitigate respiratory harmonic interference. Adaptive filtering and empirical mode decomposition-based methods attempt to separate heartbeat and respiratory components in the time-frequency domain [20,21], but their effectiveness degrades when harmonic frequencies overlap with the cardiac band. Variational mode extraction techniques, such as the adaptive separate variational mode extraction method, offer improved frequency localization yet require careful parameter tuning and prior knowledge of signal components [14]. Deep neural network approaches, including phase correction networks and DNN-based heartbeat probability estimation, have shown promise in complex scenarios; however, their dependence on training data and generalization across subjects remains a concern [16,22]. Model-based estimation methods incorporating Cramer–Rao lower bound analysis have established theoretical limits on frequency estimation accuracy, confirming that the signal-to-interference ratio is the key factor limiting heart rate measurement [11,22]. Despite these advances, existing techniques still lack a systematic framework that jointly addresses signal enhancement, harmonic identification, and robust heart rate estimation without extensive calibration or subject-specific training.

To overcome these limitations, we propose a three-stage signal processing framework for FMCW radar-based heart rate monitoring. First, a slow-time phase correlation method enhances the physiological signal by coherently aggregating energy from neighboring range bins that exhibit high phase correlation with the chest reference pixel. Second, complementary ensemble empirical mode decomposition is applied to decompose the enhanced signal, followed by an adaptive harmonic matching filtering procedure that identifies and eliminates respiratory harmonic components from the cardiac signal. Third, heart rate is estimated using autocorrelation, which provides robust periodicity measurement in the cleaned signal. The proposed method operates without requiring training data, subject-specific calibration, or prior knowledge of the harmonic structure. We validate the framework through experiments conducted with a 77 GHz FMCW radar and demonstrate that heart rate can be estimated with less than 5% error relative to a wearable reference sensor under respiratory harmonic interference.

The remainder of this paper is organized as follows. Section 2 presents the basic principles and system modeling of non-contact vital sign monitoring based on FMCW radar. The signal processing framework, the proposed methods, and simulation analysis are introduced in Section 3. Section 4 provides experimental results, followed by the Section 5 and Section 6.

## 2. Signal Model

**Figure 1:**
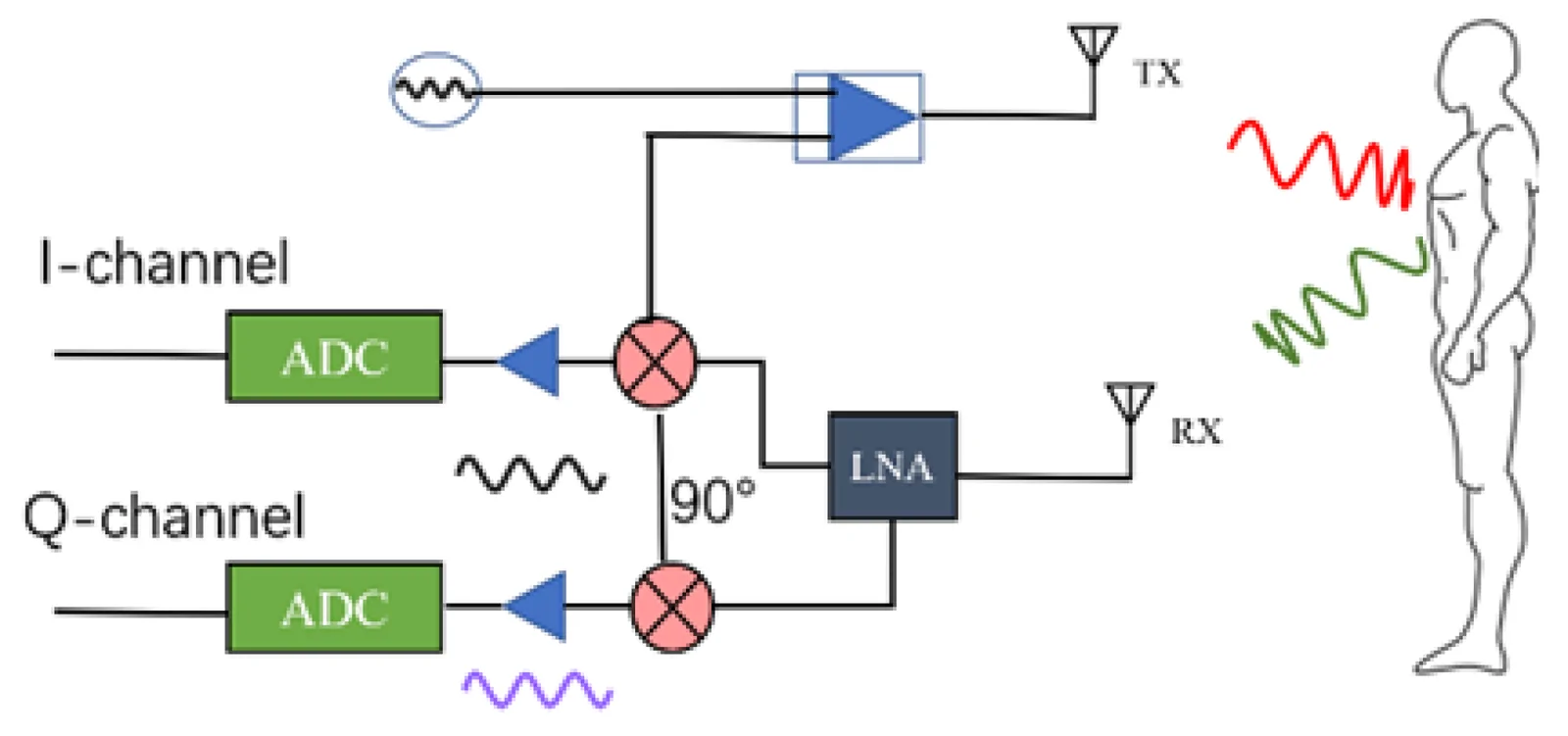
Diagram of vital signs monitoring system.

Figure 1 shows the basic operational principle of contactless radar-based vital sign monitoring. Millimeter-wave FMCW radar transmits a frequency-modulated continuous signal whose transmitted waveform can be expressed as

(1) $$x_{T}\left(t\right)=A_{T}exp\left[j\left(2\pi f_{c}t+\pi {Kt}^{2}+\phi \left(t\right)\right)\right]$$

where $A_{T}$ is the amplitude of the transmitted signal, $f_{c}$ is the operating frequency (the initial frequency of chirp), K is the frequency modulation slope, K=B/T, B is the pulse bandwidth, T is the pulse duration, ϕ(t) is the phase noise (local oscillator) of the transmitter. The phase information of the echo signal includes the movement of the chest wall. The echo signal received from the antenna RX is a delayed version of the transmitted signal, and the transmitted FMCW signal can be written as

(2) $$x_{R}\left(t\right)=A_{T}exp\left[j\left(2\pi f_{c}\left(t-t_{d}\right)+\pi K{\left(t-t_{d}\right)}^{2}+\phi \left(t-t_{d}\right)\right)\right]$$

where $t_{d}=2d/c$ is the time delay of the transmitted signal reflected by the target at a distance d from the radar, and c is the speed of light. After the received signal and the transmitted signal are orthogonally mixed and frequency down-converted, a fixed frequency intermediate frequency (IF) single-tone signal is obtained, which can be approximated as

(3) $$\mathrm{IF}=A_{\Psi }exp\left[j\left(2\pi f_{\Psi }t+\psi \left(t\right)+\Delta \theta \left(t\right)\right)\right]$$

which represents the beat signal, where $A_{\Psi }$ represents the amplitude of the signal and $f_{\Psi }$ is the beat frequency, and

(4) $$\psi \left(t\right)=2\pi f_{c}t_{d}+\pi K{t_{d}}^{2}$$

where ψ(t) is the time-varying phase related to the motion of the monitored object. Δθ(t) is the residual phase noise, which is typically ignored in short-range applications due to distance-related effects [19]. Additionally, the term $\pi K{t_{d}}^{2}$ in Equation (4) can also be disregarded in practical scenarios [20]. Finally, for a measurement object at a distance $d_{0}$ from the radar, we have

(5) $$\psi \left(t\right)=\frac{4\pi }{\lambda }\left(d_{0}+d(t)\right)$$

where $\lambda =c/f_{c}$, is the starting wavelength of a frequency-modulated continuous wave and d(t) corresponds to the chest-wall movement caused by respiration and heartbeat. In this case, Equation (5) can be written as

(6) $$\psi \left(t\right)=\frac{4\pi }{\lambda }\left(d_{0}+d_{h}\left(t\right)+d_{r}\left(t\right)+d_{h\delta }\left(t\right)+d_{r\delta }(t)\right)$$

where $d_{r}\left(t\right)$ and $d_{h}\left(t\right)$ represent displacement signals caused by respiration and heartbeat, respectively. $d_{r\delta }(t)$ and $d_{h\delta }\left(t\right)$ correspond to the harmonic signals of respiration and heartbeat. The displacement in the echo information comes not only from the surface of the chest wall but also from other areas covered by the radar, such as the abdomen and shoulders. The periodicity of the chest-wall displacement signal d(t) is analyzed to estimate the frequencies of respiration and heartbeat.

In practical deployment, the received radar signal is contaminated by multipath reflections from stationary clutter and non-stationary interference sources within the radar field of view. These interference components can significantly exceed the amplitude of the cardiopulmonary displacement signals, resulting in the accurate recovery of $d_{r}\left(t\right)$ and $d_{h}\left(t\right)$ and consequently reliable vital sign estimation—a fundamentally challenging task.

## 3. Vital Signal Processing

**Figure 2:**
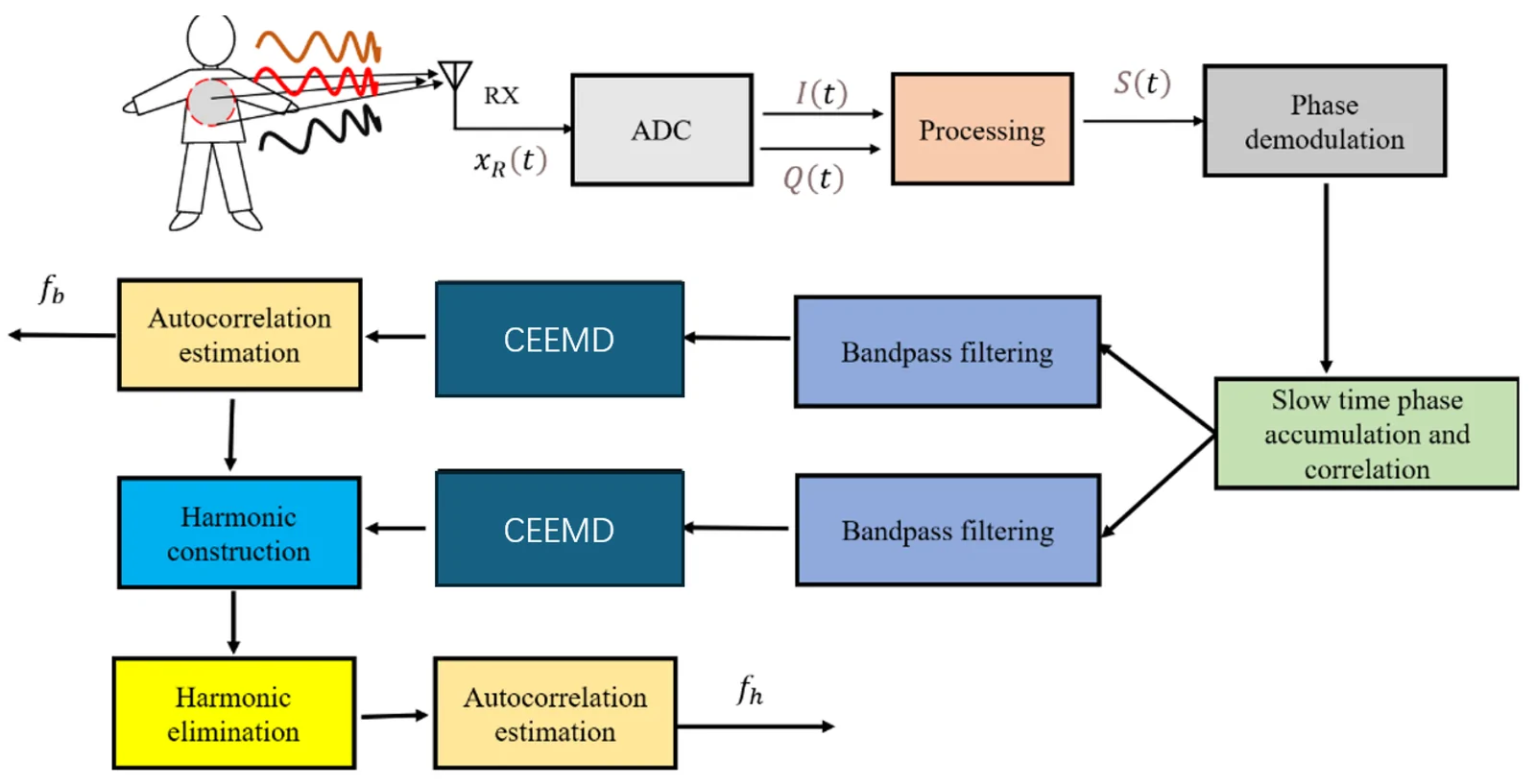
Block diagram of signal processing chain.

The proposed signal processing framework is illustrated in Figure 2. The preprocessing module receives baseband in-phase and quadrature samples I(t) and Q(t) from the analog-to-digital converter. The system was configured with a single transmitter and four receivers (single-TX, four-RX configuration). The target range-angle cell is identified as the spatial location with maximal beat-signal energy. Slow-time samples are accumulated over at least one complete respiratory cycle. Phase demodulation via phase unwrapping of the slow-time beat signal recovers the chest-wall displacement waveform.

In this work, arctan demodulation [23,24,25] is utilized for this purpose. For millimeter-wave radar signals, when the actual displacement exceeds λ/4, the phase falls within the range of [−π, π] after phase unwrapping. To address possible phase discontinuities caused by the arctan function, the extended DACM algorithm is employed to resolve the phase ambiguity issue. Due to inherent hardware defects, it is necessary to compensate for possible DC offsets before phase unwrapping. For this purpose, we employ the dynamic center tracking algorithm, which provides an optimal estimate for this problem [15,26].

After phase demodulation, the covariance correlation coefficient is computed between the displacement signal at the reference range bin (the bin with highest chest reflectivity) and signals at adjacent range bins. Pairs whose correlation exceeds a predefined threshold share consistent frequency and phase characteristics and are coherently summed to enhance the signal-to-noise ratio (SNR). The enhanced signal is then bandpass-filtered to remove residual DC drift and high-frequency noise.

Bandpass filtering isolates the respiratory band (0.1–0.8 Hz, corresponding to 6–48 breaths per minute) and the cardiac band (0.8–3 Hz, corresponding to 48–180 beats per minute) [27,28]. These ranges encompass both the fundamental frequencies and their physiologically relevant harmonics.

The bandpass-filtered signals are independently processed by complementary ensemble empirical mode decomposition (CEEMD) to obtain purified respiratory and heartbeat components in the time-frequency domain. The respiratory rate is then estimated via autocorrelation. However, the heartbeat signal after CEEMD may still contain residual respiratory harmonics. To address this, a harmonic matching and subtraction procedure is applied to construct and remove the respiratory harmonic interference, after which heart rate is estimated using autocorrelation.

### 3.1. Baseband Signals

The received signal after orthogonal mixing yields I/Q baseband signals:

(7) $$I\left(t\right)=A_{I}cos\left(2\pi f_{\Psi }t+\frac{4\pi }{\lambda }\psi \left(t\right)+\Delta \theta \left(t\right)\right)+{DC}_{I}$$

(8) $$Q\left(t\right)=A_{Q}sin\left(2\pi f_{\Psi }t+\frac{4\pi }{\lambda }\psi \left(t\right)+\Delta \theta \left(t\right)\right)+{DC}_{Q}$$

where $A_{I}$ and $A_{Q}$ represent the amplitude of in-phase and quadrature signals, while $\;{DC}_{I}$ and ${DC}_{Q}$ denote the direct current offset of the orthogonal baseband signals. There are two main sources of this DC offset. One is the reflection from fixed clutter, and the other is the accumulated DC offset in the RX link [27]. To correct DC offset, this article utilizes the dynamic center tracking algorithm. The algorithm offers maximum likelihood estimation for the offset of in-phase and quadrature signals, centered at the origin [28,29].

### 3.2. Signal Enhancement

The wide bandwidth of FMCW millimeter-wave radar enables fine range resolution. For a 77 GHz radar with 4 GHz bandwidth, the range resolution is approximately 4 cm. At this resolution, the echo from a human torso is a superposition of returns from multiple body regions, each exhibiting slightly different displacement patterns due to respiration and cardiac activity.

To exploit the spatial diversity of these returns, we propose a slow-time phase correlation processing method. The Pearson correlation coefficient between the displacement signals from two range bins is computed as

(9) $$\xi_{correlation}=\frac{\sum_{i=1}^{n}\;\left(x_{i}-\bar{x}\right)\left(y_{i}-\bar{y}\right)}{\sqrt{\sum_{i=1}^{n}\;{\left(x_{i}-\bar{x}\right)}^{2}\sum_{i=1}^{n}\;{\left(y_{i}-\bar{y}\right)}^{2}}}$$

where $x_{i}$ and $y_{i}$ represent the discrete values of signals from different regions. The variable i denotes the discrete point, with *i* ranging from 1 to *N*. $\bar{x}$ and $\bar{y}$ represent the means of signals $x_{i}$ and $y_{i}$, respectively. The term “two range bins” refers to two adjacent range FFT bins (i.e., spatial range cells after the range FFT operation). The selection criterion for choosing the specific bins is based on the energy peak in the range profile: the bin with maximum energy is selected as the reference bin, and its immediately neighboring bin (adjacent in the range dimension) with the highest correlation coefficient (threshold: >0.7) to the reference bin is selected as the secondary bin for coherent aggregation.

**Figure 3:**
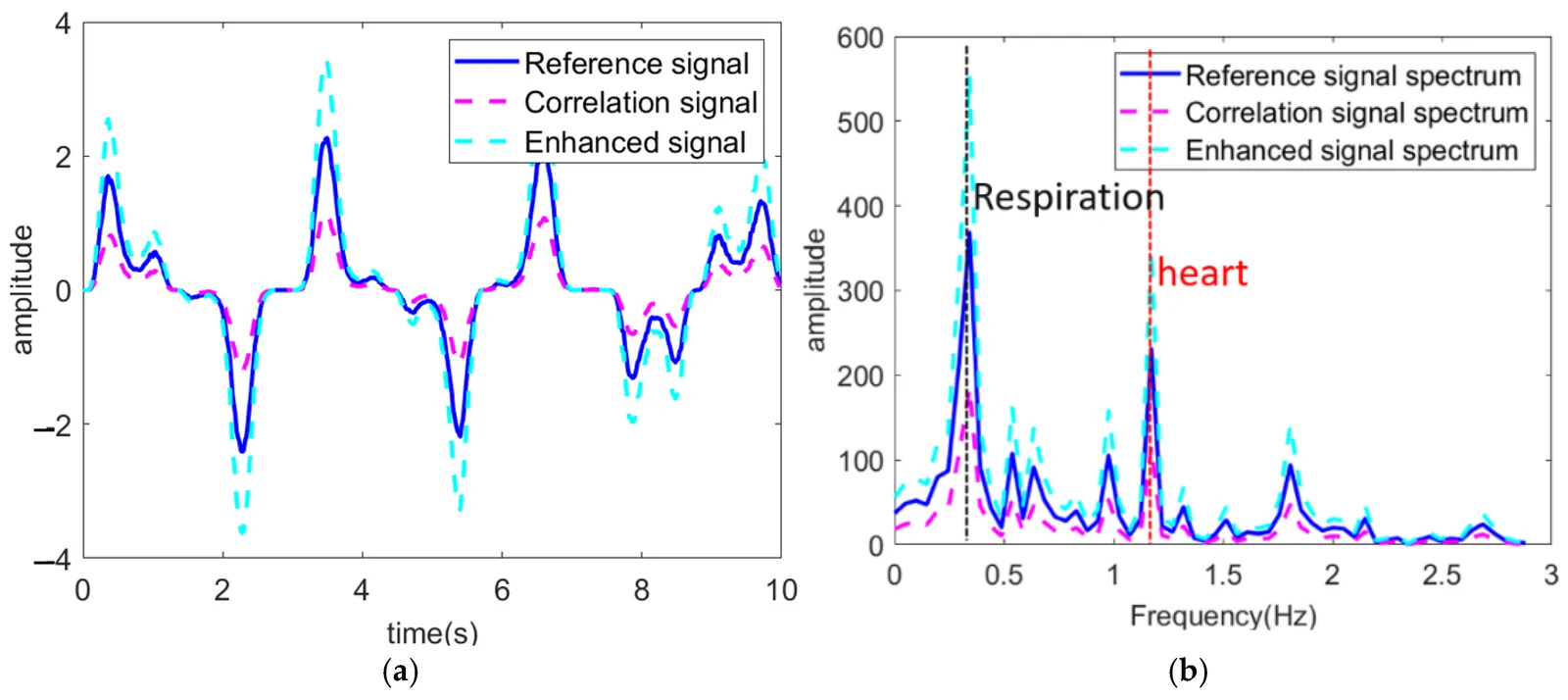
Simulated breathing and heartbeat superimposed signals. (**a**) Reference signal, correlation signal and enhanced signal. (**b**) Signal spectrum.

Figure 3 illustrates the phase correlation between two adjacent range bins. The two displacement signals shown are the phase-demodulated chest-wall displacement signals extracted from range bin k (the reference bin with maximum energy) and range bin k + 1 (its immediate neighbor). Both signals were acquired simultaneously from a single human subject seated at 0.6 m from the radar during normal breathing. The respiration-dominated low-frequency trend and the superimposed heartbeat-induced micro-displacements are visible in both signals. The Pearson correlation coefficient was calculated over the full acquisition window using the standard formula $\xi_{correlation}$, yielding a value of 0.93 (Figure 3a) for the representative pair shown, indicating strong linear correlation between the two adjacent range-bin signals. After superposition, the signal amplitude was enhanced while the frequency content remained unchanged (Figure 3b), confirming that coherent energy aggregation preserves the spectral characteristics of the vital signs. The same threshold-based correlation procedure was applied to experimental data, as described below.

### 3.3. CEEMD-Based Signal Decomposition

This order-of-magnitude amplitude disparity causes respiratory harmonics to match or exceed the cardiac fundamental in the displacement spectrum. Conventional frequency-domain methods therefore struggle to isolate the heartbeat component. CEEMD offers a data-driven approach that decomposes non-stationary signals into intrinsic mode functions (IMFs) based on local time-frequency characteristics [30]. However, CEEMD alone cannot separate components whose frequencies are closely spaced. In this work, CEEMD is used to separate harmonics with distinctly different frequencies from the heartbeat band; residual overlapping harmonics are handled by the subsequent harmonic matching stage.

Let the bandpass-filtered respiratory and heartbeat signals be denoted in the frequency domain as

(10) $$\psi_{r}\left(f\right)=A_{r}T\cdot \mathrm{sinc}\left(T\left(f-f_{r}\right)\right)+∆\varphi_{r}\left(f\right)$$

(11) $$\psi_{h}\left(f\right)=A_{h}T\cdot \mathrm{sinc}\left(T\left(f-f_{h}\right)\right)+∆\varphi_{h}\left(f\right)$$

where $A_{r}$ is the amplitude of the respiratory signal, $A_{h}$ is the amplitude of the heartbeat signal, T is the observation window length, $f_{r}$ is the frequency of the respiratory signal, $f_{h}$ is the frequency of the heartbeat signal, and $∆\varphi_{r}$ and $∆\varphi_{h}$ are the noise signal spectra of the respiratory and heartbeat signals, respectively. These signals consist of harmonic noise and other high-frequency noise.

Assuming x(n), n = 1, 2, …, *N*, is the discrete expression of the extracted signal. ${IMF}_{j}$ represents the j-th IMF output obtained by the method in this paper, and ${EMD}_{j}\left(\cdot \right)$ represents the j-th mode obtained by EMD. The calculation process of the method in this paper is as follows:

(1) Generate L realizations of the signal with added positive and negative Gaussian white noise:

(12) $$x_{i}^{+}\left(n\right)=x\left(n\right)+w^{i}\left[n\right]i=1,2\ldots \ldots \ldots L$$

(13) $$x_{i}^{-}\left(n\right)=x\left(n\right)-w^{i}\left[n\right]i=1,2\ldots \ldots \ldots L$$

(2) Decompose each noisy realization using EMD to obtain IMF components:

(14) $${IMF}_{j}\left(n\right)=\frac{1}{L}\sum_{i=1}^{L}\left(\left[{EMD}_{j}\left(x_{i}^{+}\left(n\right)\right)+{EMD}_{j}\left(x_{i}^{-}\left(n\right)\right)\right]/2\right)$$

(3) Compute the residue after extracting the j-th IMF:

(15) $$r_{j}\left(n\right)=r_{j-1}\left(n\right)-{IMF}_{j}\left(n\right)$$

where $r_{0}\left(n\right)=x\left(n\right)$.

**Figure 4:**
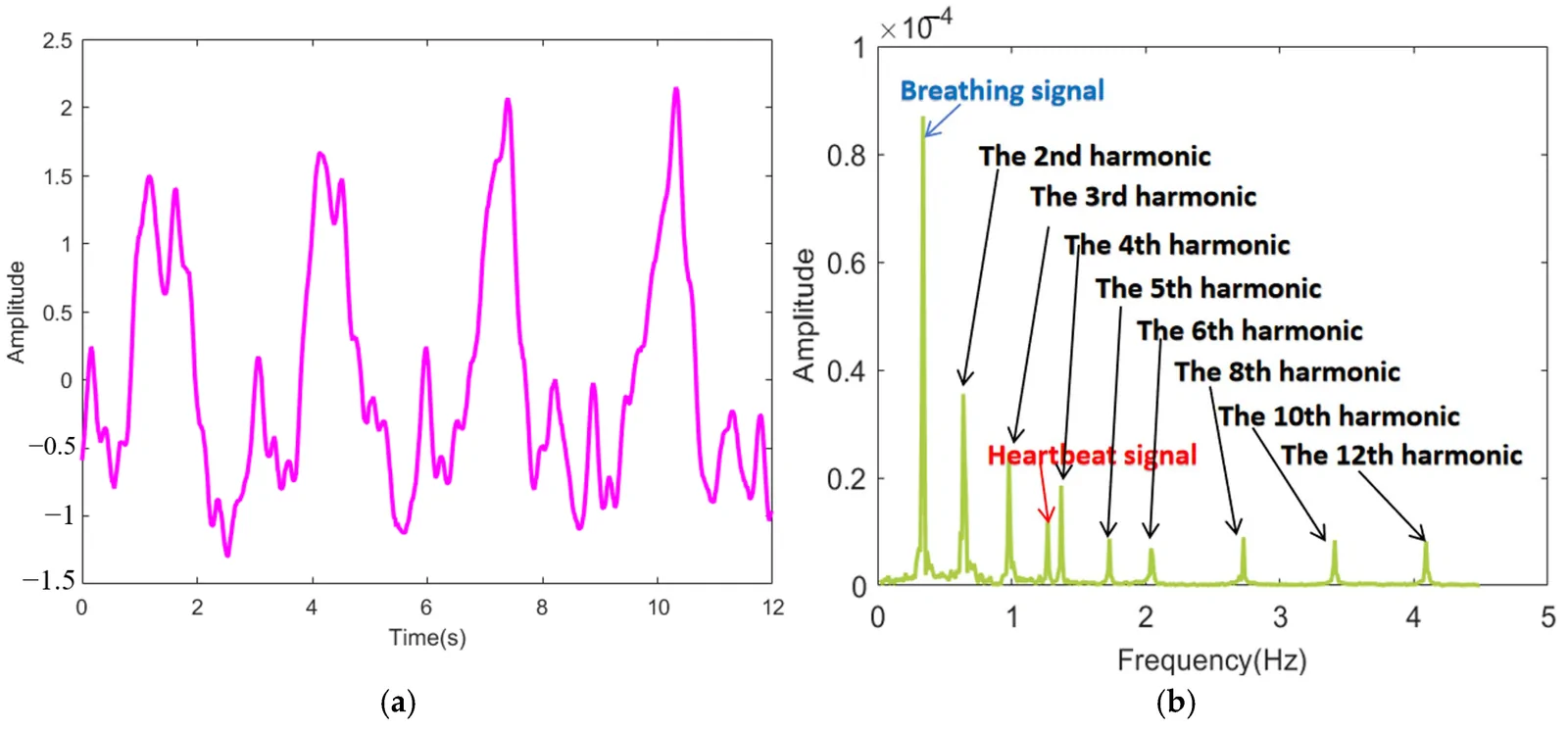
Simulation signal (**a**) and its corresponding spectrum graph (**b**).

To improve the efficiency and reduce the decomposition time of the CEEMD algorithm, the decomposition process is halted when the frequency of an IMF component falls below 0.2 Hz. In order to demonstrate the effectiveness of the proposed method in separating the heartbeat signal and respiratory harmonics, Figure 4a showcases a simulated signal that consists of intertwined heartbeat signal and respiratory harmonics. Figure 4b displays the corresponding spectrogram. The parameters of the simulated signal are determined using Equations (10) and (11), with $f_{r}$ = 20 bpm (0.333 Hz) and $f_{h}$ = 73 bpm (1.27 Hz). The amplitudes of the respiratory harmonics are set as follows: |$A_{r}1$|:|$A_{r}$2|:|$A_{r}$3|:|$A_{r}$4|:|$A_{r}$5|:|$A_{r}$6|:|$A_{r}$8|:|$A_{r}$10|:|$A_{r}$12|:|$A_{h}$| = 10:5:3:2:1:1:1:1:1:1.4. The corresponding phases are randomly selected. The sampling rate is set to 50 Hz.

To automatically identify the IMFs corresponding to respiration and heartbeat, the amplitude and frequency bounds ($A_{min}$, $A_{max}$, $f_{min}$, $f_{max}$) are defined for each physiological signal according to Table 1. An IMF is considered a candidate if its dominant amplitude and frequency fall within the respective bounds. Among all candidates, the IMF with the highest amplitude within the valid frequency range is selected as the final respiratory or cardiac component.

**Figure 5:**
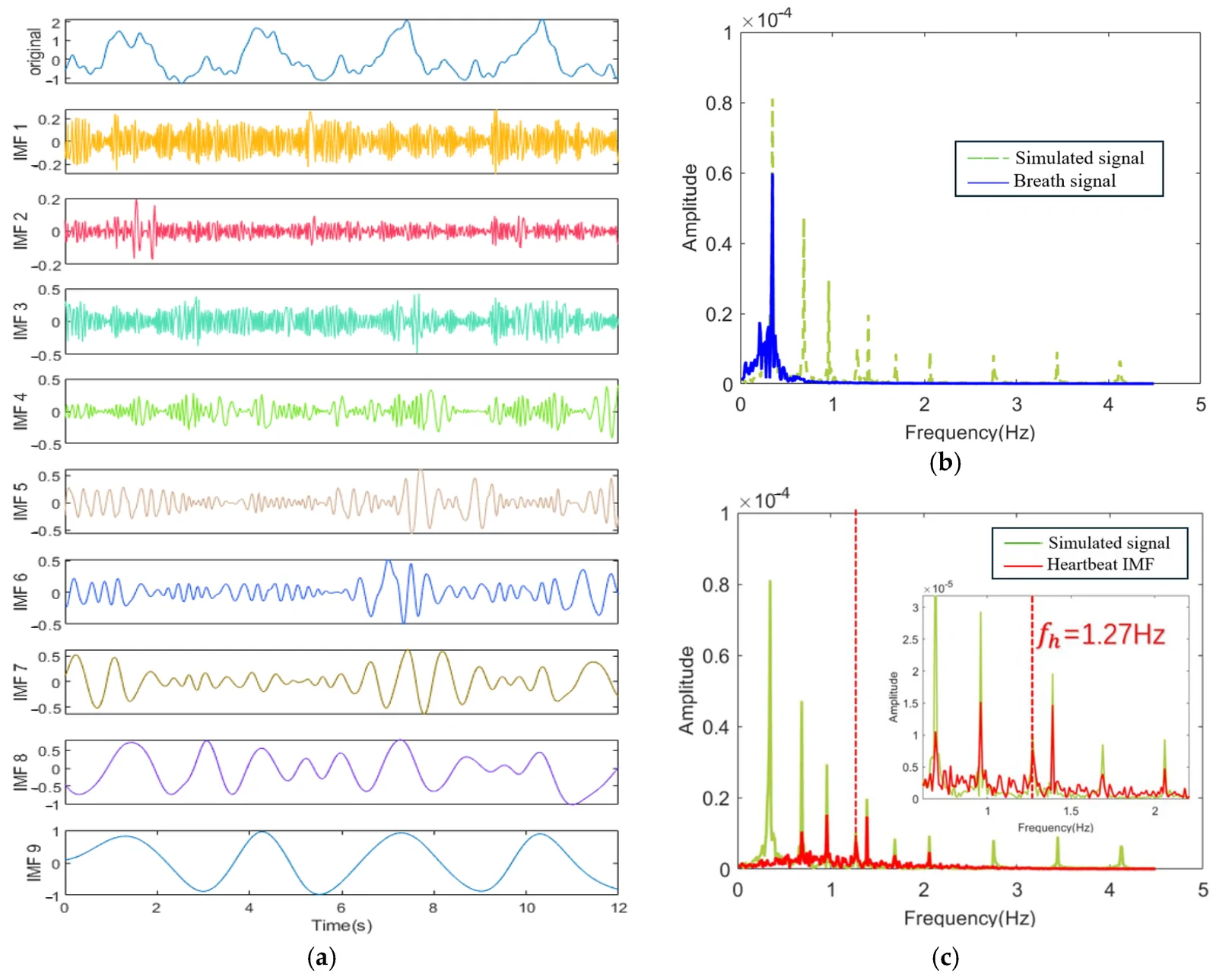
(**a**) shows the improved CEEMD results of the analog signal application, (**b**) displays the frequency spectrum of the analog signal and the respiratory IMF, and (**c**) shows the frequency spectrum of the analog signal and the heartbeat IMF. The respiratory and heartbeat frequencies are set to the same values as in Figure 4, i.e., $f_{r}$ = 0.333 Hz and $f_{h}$ = 1.27 Hz. IMF9 and IMF7 are identified as the closest components to the respiratory and heartbeat, respectively.

Figure 5 shows the IMF components of the heartbeat obtained by the method described in this article, as well as the spectrograms of the respiratory component and the heartbeat component. The parameter settings are the same as those in Figure 4. From Figure 5a, it can be observed that the respiratory component is the 9th IMF and the heartbeat component is the 7th IMF. The corresponding spectrograms of the respiratory component and the heartbeat component are shown in Figure 5b and Figure 5c respectively. It can be seen that the respiratory frequency can be easily estimated using DFT from the respiratory component; however, the heartbeat component contains multiple respiratory harmonics, and existing methods cannot distinguish between the heartbeat component and respiratory harmonics.

**Figure 6:**
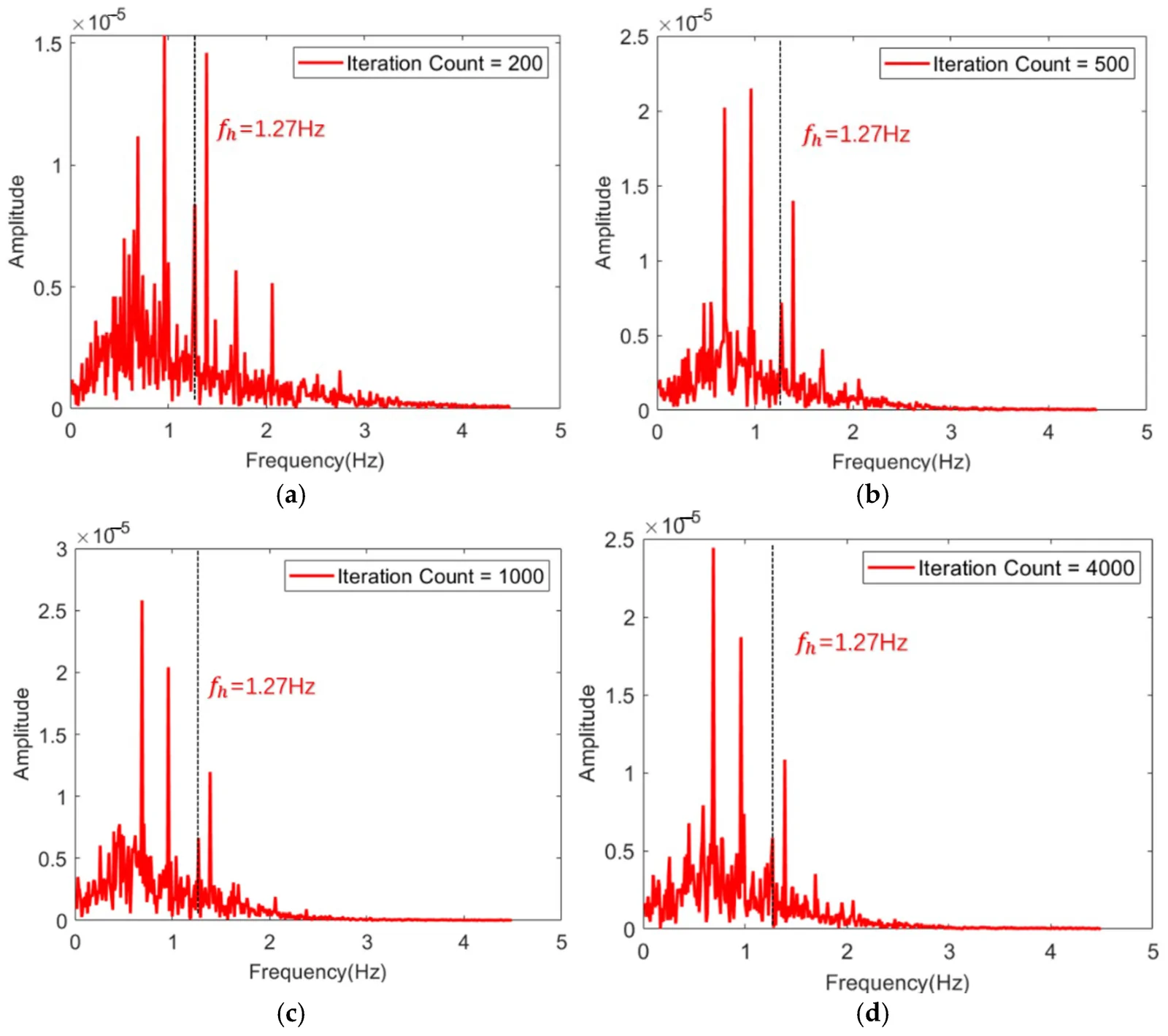
Spectrum plots of heartbeat IMF obtained at different iteration counts: (**a**) 200 iterations, (**b**) 500 iterations, (**c**) 1000 iterations, and (**d**) 4000 iterations. The dashed lines in the figure denote heart-rate frequency points.

Figure 6 shows a comparison of the spectra of heartbeat intrinsic mode functions (IMFs) obtained using our proposed method at different iteration numbers. The parameter settings are the same as in Figure 4. The results in Figure 6a–d are from the same segment of the simulated signal. From Figure 6, it can be seen that the improved CEEMD method effectively suppresses high-order harmonics and some low-order harmonics of respiration when obtaining the spectrum of the heartbeat component. However, for respiration harmonics close to the heartbeat frequency, changing the iteration number can achieve some degree of suppression, but complete elimination of harmonic interference is not possible. This limitation is one of the reasons for the reduced accuracy in heart rate estimation in existing methods.

### 3.4. Harmonic Construction and Elimination

The periodicity of respiration and heartbeat allows their displacement waveforms to be modeled as a Fourier series. The composite displacement signal can be expressed as a superposition of the respiratory fundamental, respiratory harmonics, heartbeat fundamental, and heartbeat harmonics:

(16) $$s_{M}\left(n\right)=\sum_{\xi =1}^{\xi_{\zeta }}\;c_{\zeta }exp\left(j\omega_{\zeta }\xi n\right)\;$$

where $s_{M}\left(n\right)$ is the discrete form of the echo for nϵ{1,2,3…N}, $c_{\zeta }$ is the complex amplitude of the ξ harmonic. The angular frequency $\omega_{\zeta }$ can be calculated using the formula $\omega_{\zeta }=2\pi f_{\xi }$, $f_{\xi }$ can be determined using the formula $f_{\xi }=1/\tau_{\xi }$, where $\tau_{\xi }$ is the period of respiration. The value of $c_{\zeta }$ can be expressed as

(17) $$c_{\zeta }={\left[A_{1}exp\left(j\varphi_{1}\right),A_{2}exp\left(j\varphi_{2}\right)\ldots ,A_{\zeta }exp\left({j\varphi }_{\zeta }\right)\right]}^{T}$$

Let $\Phi_{\zeta }\;$ denote the matrix whose columns are the fundamental and harmonic basis vectors of the respiratory signal. $\Phi_{\zeta }\;$ is composed of M complex sinusoids at various frequencies:

(18) $$\Phi_{\zeta }=\left[\Phi \left(f_{r}\right)\Phi \left(f_{r.2}\right)\ldots \Phi \left(f_{r.3}\right)\ldots \Phi (f_{r.\xi_{\zeta }})\right]\;$$

where $\Phi (f_{r})={\left[e^{j2\pi f_{r}*1},e^{j2\pi f_{r}*2}\ldots ,e^{j2\pi f_{r}*(N)}\right]}^{T}$. Then (16) can be rewritten as

(19) $$s_{M}\left(n\right)=\Phi_{\zeta }c_{\zeta }\;$$

Minimize the sum of squared residuals between the echo and signal models to obtain $c_{\zeta }$. This can be described as follows:

(20) $$min\;\varepsilon ={\left|F\left(n\right)-s_{M}\left(n\right)\right|}^{2}$$

where F(n) is the overlay of signals in the heartbeat region after simplification, as reported in [17]. In this paper, the $s_{M}\left(n\right)$ signal is obtained by gradient descent algorithm. The optimal values for the coefficients of higher-order functions are obtained as

(21) $$c_{\zeta }={\left(\Phi_{\zeta }^{H}\Phi_{\zeta }\right)}^{-1}\Phi_{\zeta }^{H}F\left(n\right)$$

To enhance computational efficiency, the initial parameters are set based on empirical values. Specifically, the initial value of ε is established within the range of 3/20 to 1/5 of the respiratory signal’s amplitude, while the initial amplitude for respiratory harmonics is determined to be 1/15 of the respiratory signal’s amplitude. Respiration rate and heart rate are estimated every 10 s, so there is enough time to get the $c_{\zeta }$.

The CEEMD identifies the IMF containing the respiratory fundamental. Once the respiratory and cardiac IMFs are identified, further decomposition is terminated.

By obtaining the optimal values of each coefficient, we can evaluate the optimal fundamental frequency and its harmonics that occupy the respiratory signal spectrum. This enables us to accurately assess the respiratory rate. As mentioned in reference [14], the third to fifth order respiratory harmonics can have a significant impact on the heartbeat signal, hence ξ=3 and $\xi_{\zeta }$ = 5. Effectively determining these parameters reduces the computational burden of grid searching.

**Figure 7:**
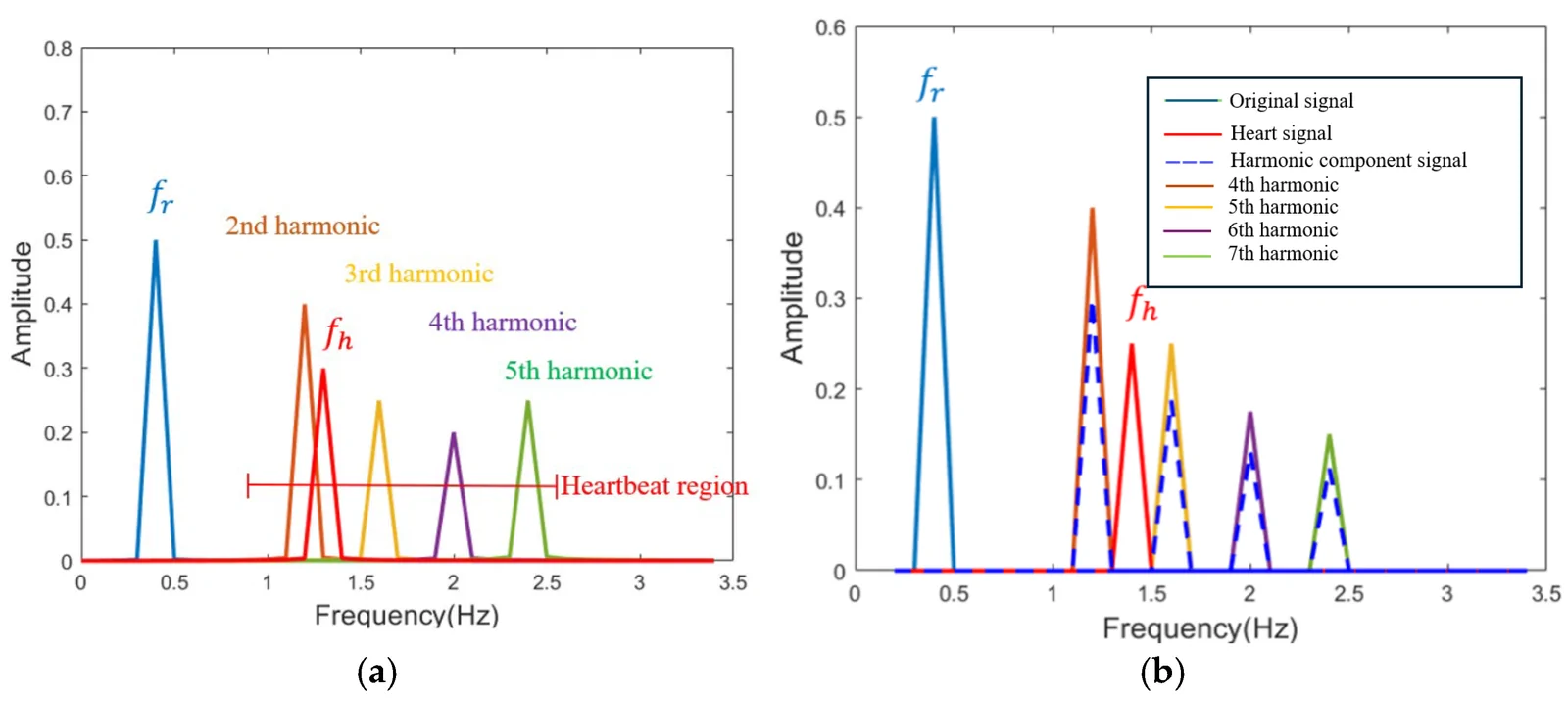
Displacement signal simulation: (**a**) the spectrum of simulated heartbeat and breathing signals; (**b**) harmonic signal spectrum constructed from simulated signal.

Figure 7a shows the spectrum of a simulated displacement signal with $f_{r}$ = 0.4 Hz and $f_{h}$ = 1.4 Hz. The third through fifth respiratory harmonics fall within the cardiac band, and their amplitudes can exceed that of the heartbeat fundamental. Conventional peak-picking after bandpass filtering therefore cannot reliably distinguish the heartbeat from respiratory harmonics, motivating the combined CEEMD and harmonic matching approach proposed herein. As shown in Figure 7b, the blue dashed line represents the matched harmonic signal constructed based on the respiratory fundamental frequency and the simulated displacement signal. The displacement signal is first passed through a bandpass filter and CEEMD processing, and then the matched harmonic signal is removed. We can obtain a spectrum that is pure for the heartbeat signal. At this point, we have eliminated the interference of noise and respiratory harmonics, and obtained the estimated value that is closest to the actual heart rate.

Impact of the Number of Samples (*N*): The number of slow-time samples *N*, which corresponds to the duration of the acquisition window, directly affects the performance of the proposed method. A larger *N* provides finer frequency resolution ($∆f=f_{s}/N$, where $f_{s}$ = 20 Hz), which improves the accuracy of respiratory harmonic frequency estimation and the subsequent matching process. For a 10 s window (*N* = 200), the frequency resolution is 0.1 Hz, which is sufficient to resolve the respiratory fundamental (0.2–0.4 Hz) and its harmonics. A shorter window (3 s, *N* = 60) yields a coarser resolution of 0.33 Hz, which may degrade the harmonic subtraction accuracy. Conversely, increasing *N* beyond 200 (30 s) provides diminishing returns as the subject’s physiological state may drift. In our experiments, *N* = 200 (10 s window) was selected as a practical trade-off between frequency resolution and temporal stationarity.

## 4. Experimental Results

We validated the proposed framework using a 77 GHz FMCW radar (Texas Instruments AWR1443, Dallas, Texas, USA) with 4 GHz bandwidth operating in the 77–81 GHz frequency range. The system was configured with a single transmitter and four receivers (single-TX, four-RX configuration). The chirp duration was 64 µs with a 50 ms frame interval, yielding a 20 Hz slow-time sampling rate. The number of active transmitters and receivers used in the reported experiments was one transmitter and four receivers. Data processing in this paper was performed using MATLAB v2022b.

**Figure 8:**
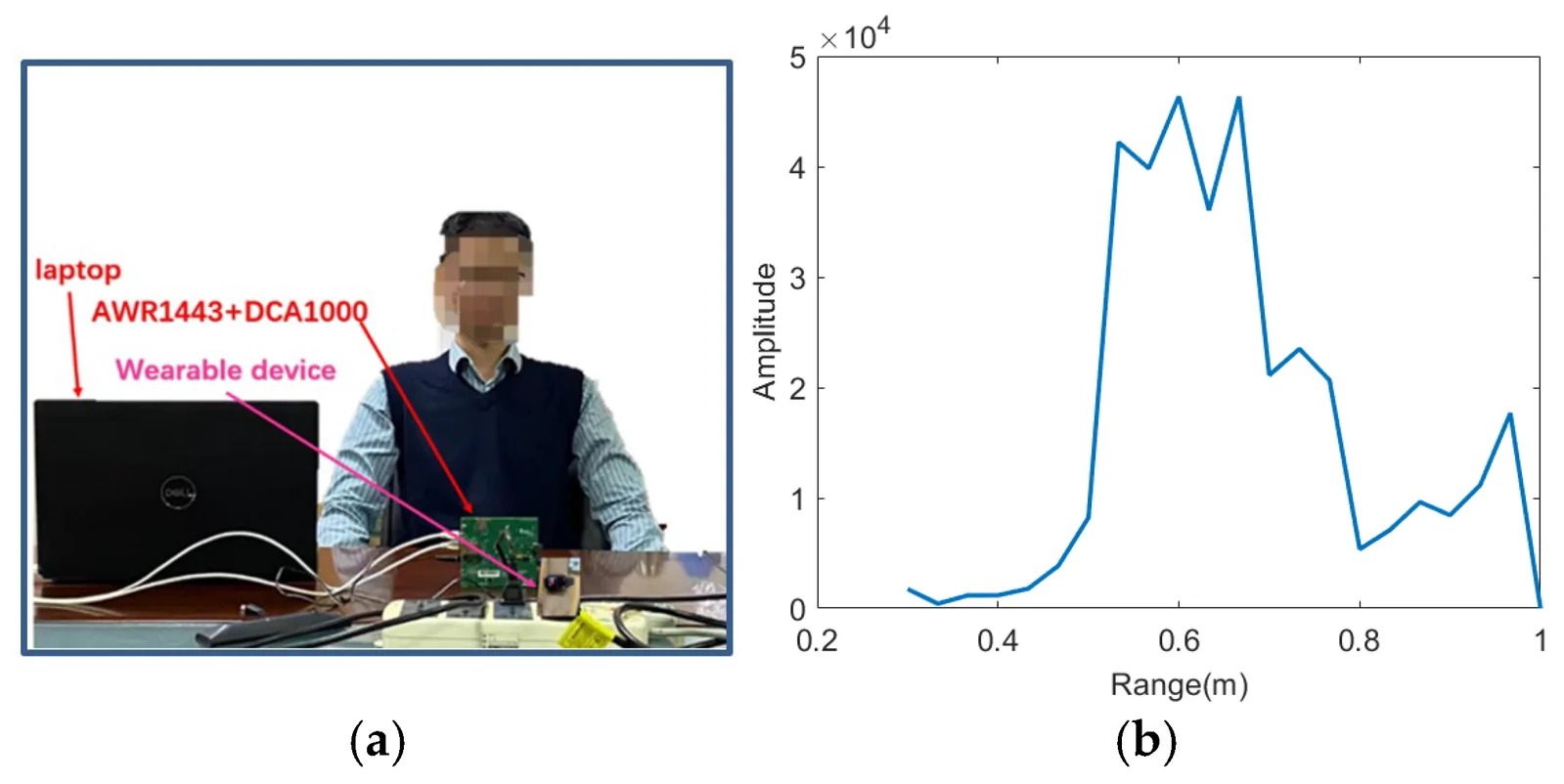
Experimental scenario: (**a**) actual test environment; (**b**) distance between human body and radar.

Figure 8a shows the measurement setup, with the subject being a male seated in front of the radar at a distance of approximately 0.6 m, instructed to breathe normally. A commercially available wearable device called MI7 was used as a reference for measuring the actual heart rate. The reference device, branded as MI7 (a commercial smart band manufactured by Xiaomi Technology Co., Ltd., Beijing, China), is a wrist-worn photoplethysmography (PPG)-based wearable sensor. It measures volumetric changes in blood flow via an optical sensor (green LED) at the wrist to derive beat-to-beat intervals, from which the heart rate is estimated. The MI7 device outputs heart rate estimates at a 1 Hz update rate (sampling rate of the HR output) and has a claimed accuracy of ±5% in steady-state conditions according to the manufacturer’s specifications. During our experiments, the MI7 was worn on the subject’s left wrist simultaneously with the radar recording, and the recorded HR values were averaged over each 10 s epoch to match the radar’s update interval.

Figure 8b illustrates the distance profile of the subject. The energy is mainly concentrated in the detected distance bin, but due to the high distance resolution, the energy also spreads to neighboring distance bins. This enables us to improve the signal-to-noise ratio by adding the energy of neighboring signals with high correlation, as described in Section 3.2.

### 4.1. Signal Enhancement Results

**Figure 9:**
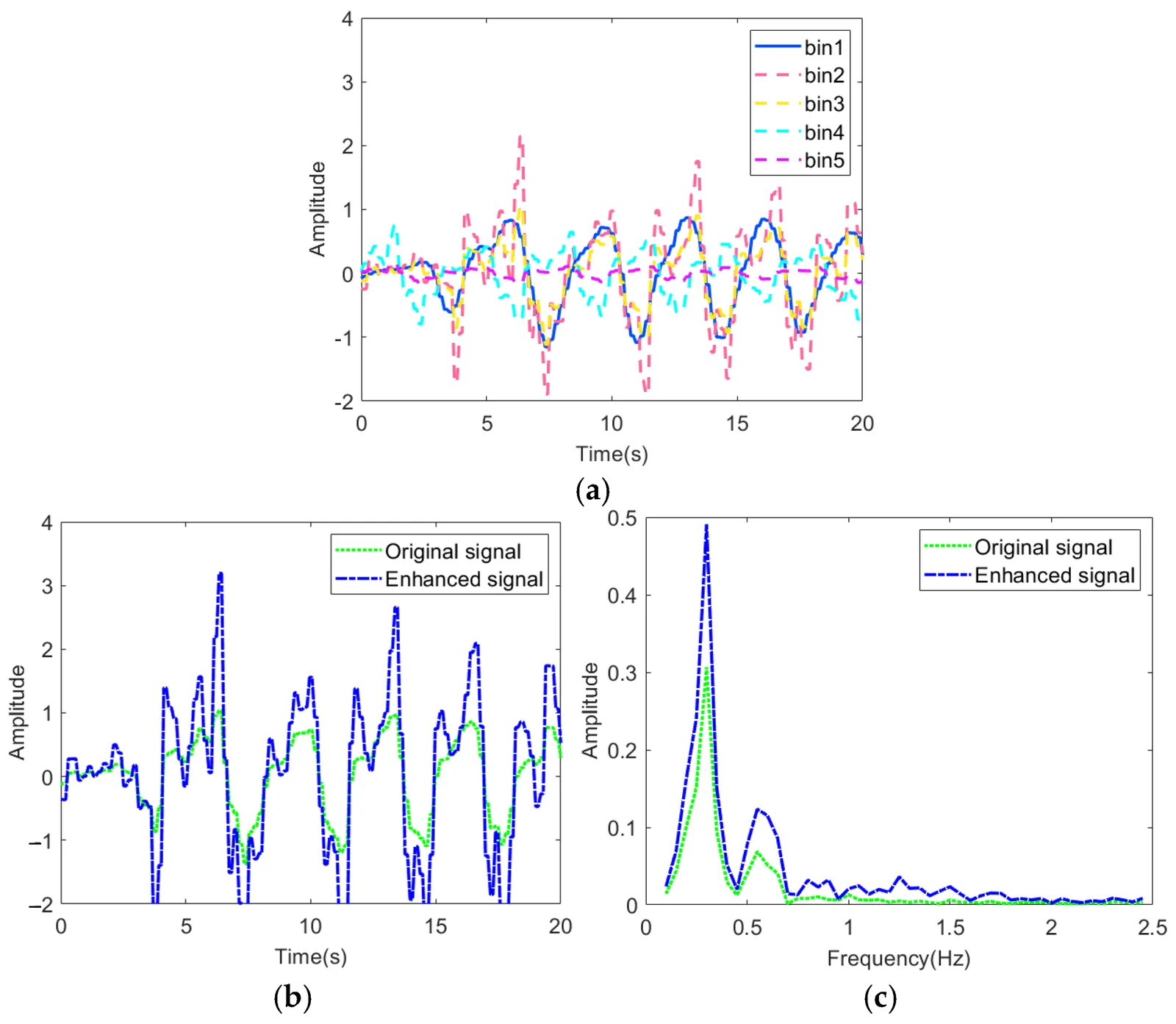
Slow-time data phase demodulation and processing. (**a**) shows the displacement signals of adjacent distance bins. (**b**) compares the original signal with the enhanced signal. (**c**) shows the spectra of the original and enhanced signals.

Phase-demodulated displacement signals from the highest-energy range bin and its four neighbors exhibited correlation coefficients of 0.90, 0.82, 0.79, 0.07, and −0.20 relative to the reference bin (Figure 9a). Bins with correlation exceeding a threshold of 0.75 were coherently summed with the reference signal. Figure 9b shows the enhanced signal obtained by coherently aggregating the reference range bin (bin 1, highest energy) with its best-correlated neighboring bin (bin 2, correlation coefficient $\xi_{correlation}$ = 0.90). The aggregation is performed by weighted averaging in the slow-time domain, with weights proportional to each bin’s correlation with the reference. After aggregation, the enhanced signal spectrum showed a marked amplitude improvement while preserving the original frequency content (Figure 9b,c), confirming that coherent summation preserves the periodic structure of both respiration and heartbeat.

### 4.2. CEEMD Results

In the absence of strong motion artifacts, the enhanced spectrum after signal enhancement was dominated by the respiratory fundamental, enabling reliable respiratory rate estimation via DFT. For heartbeat estimation, however, the signal-to-noise ratio remained insufficient and respiratory harmonics persisted. We therefore applied CEEMD to the bandpass-filtered cardiac signal to suppress out-of-band noise and higher-order harmonics.

**Figure 10:**
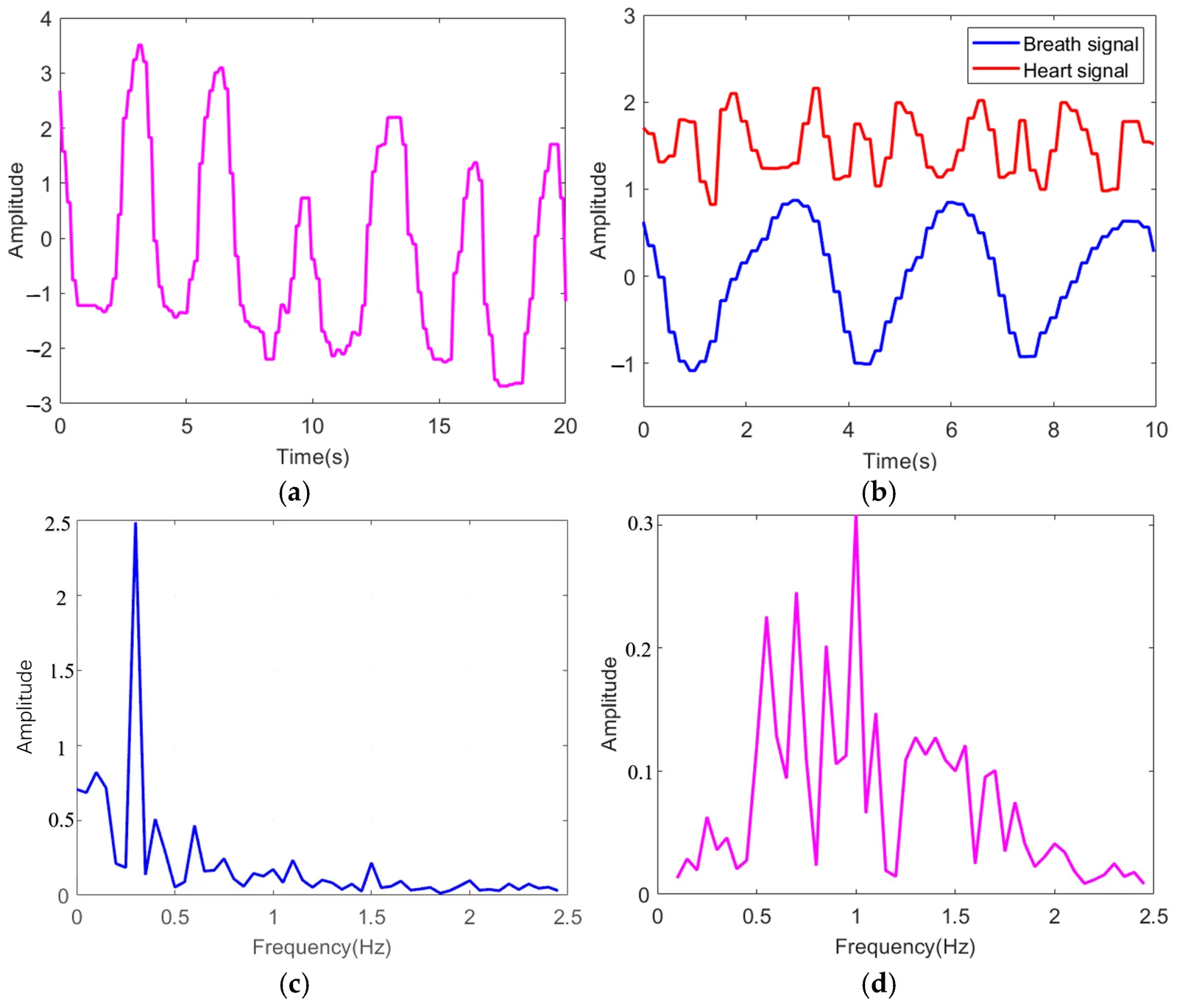
Actual test of slow-time signals: (**a**) original displacement signal; (**b**) breath signal and heartbeat signal after bandpass filtering; (**c**) respiratory signal spectrum; (**d**) heartbeat signal spectrum.

The original time-domain signal is illustrated in Figure 10a, and the time-domain signal after bandpass filtering is shown in Figure 10b. Spectrograms of the bandpass-filtered respiratory and cardiac signals are shown in Figure 10c and Figure 10d, respectively. The respiratory rate was obtained directly by spectral peak search. The cardiac spectrogram contained substantial noise and harmonic interference that precluded direct DFT-based heart rate estimation.

**Figure 11:**
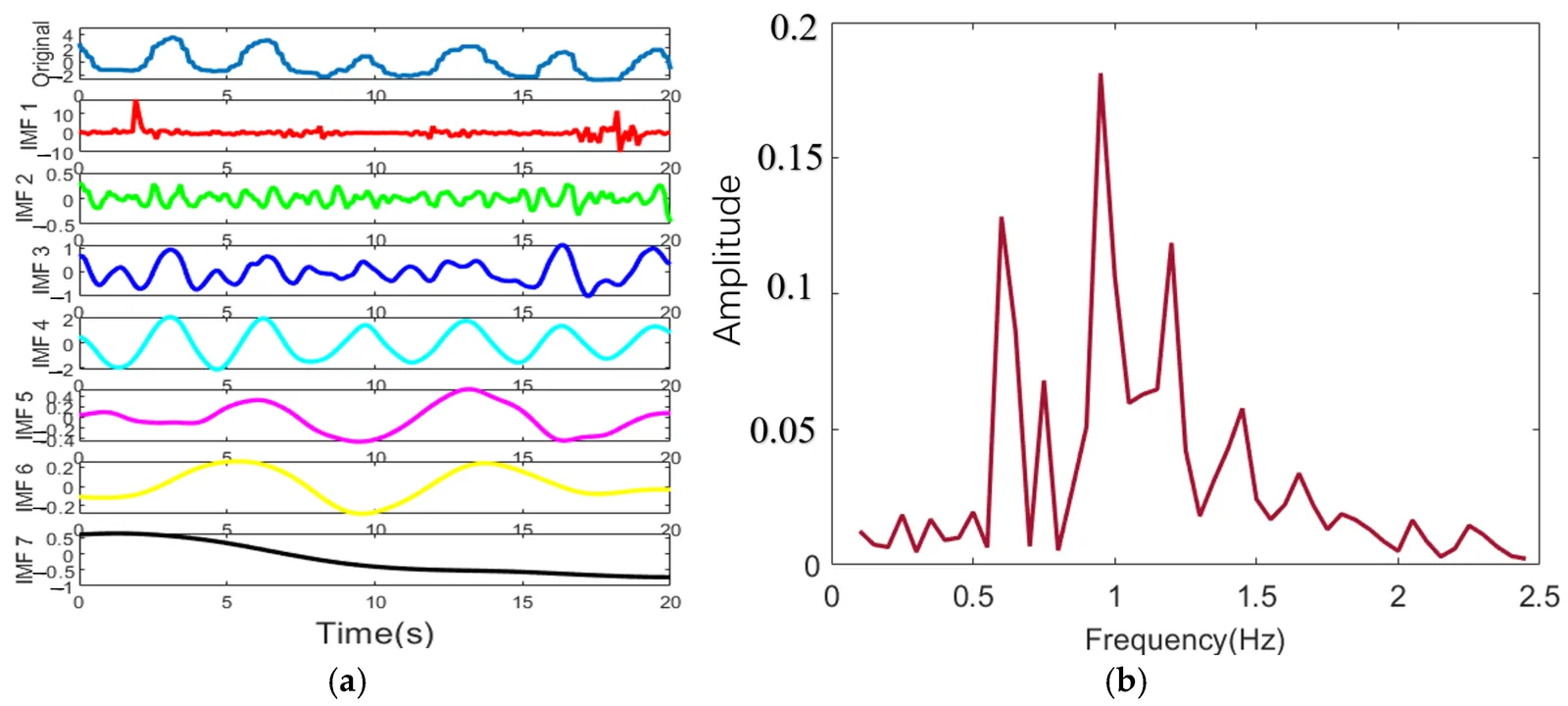
The CEEMD result of a practical signal. (**a**) The decomposition result of a practical signal using the proposed method. (**b**) The spectrograms of the practical signal, the heartbeat IMF.

CEEMD of the cardiac signal yielded multiple intrinsic mode functions (IMFs), of which the second IMF corresponded to the heartbeat component (Figure 11a). Its spectrogram (Figure 11b) showed effective suppression of broadband noise compared with the raw cardiac spectrogram. Nevertheless, residual respiratory harmonic peaks remained in the vicinity of the cardiac fundamental, necessitating the third processing stage.

### 4.3. Harmonic Elimination Results

**Figure 12:**
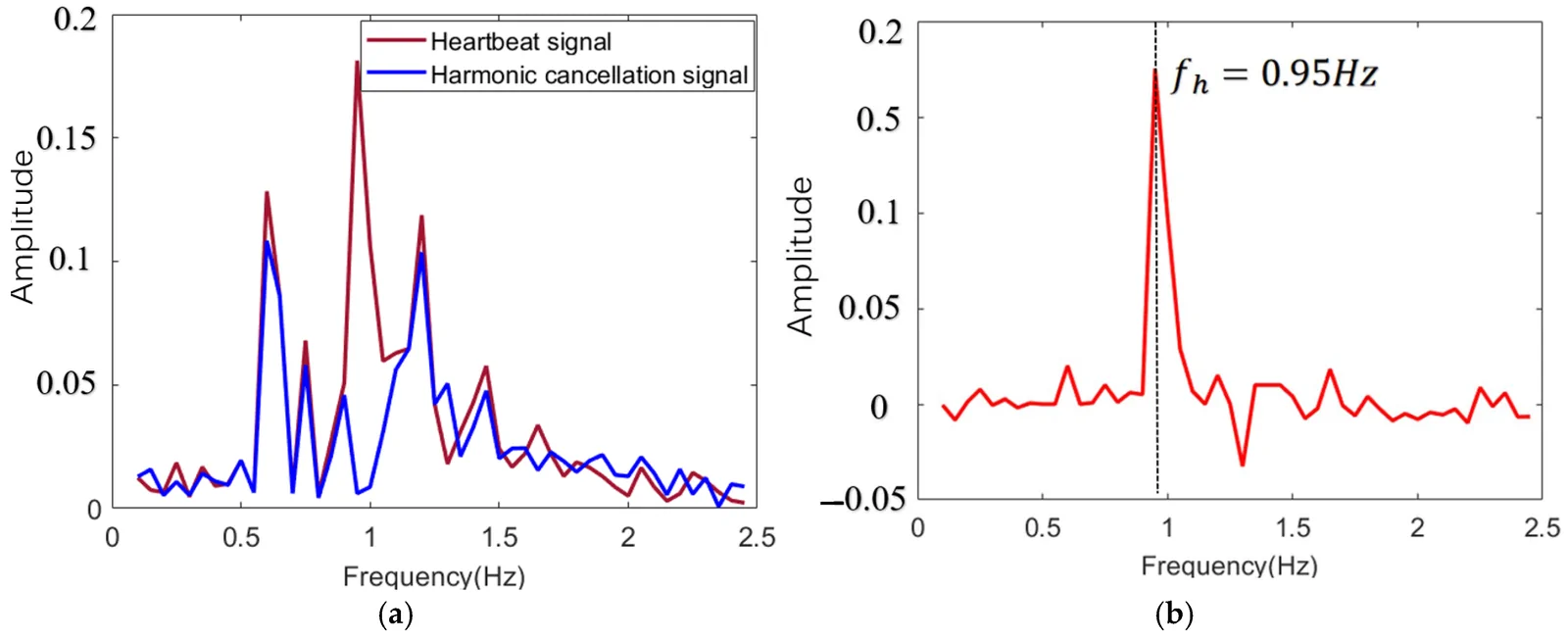
Filter out harmonic signals. (**a**) Construction harmonic signal. (**b**) The heart rate once the harmonic signal is filtered out, where the dashed lines denote heart-rate frequency points.

The harmonic matching filter constructs harmonic signals using the respiratory intrinsic mode function (IMF) obtained from CEEMD and subtracts these harmonic components from the cardiac IMF, as depicted in Figure 12a. After the subtraction operation, the residual spectrum presents only one dominant peak at the cardiac fundamental frequency (Figure 12b), which allows heart rate to be directly estimated via the autocorrelation method.

**Figure 13:**
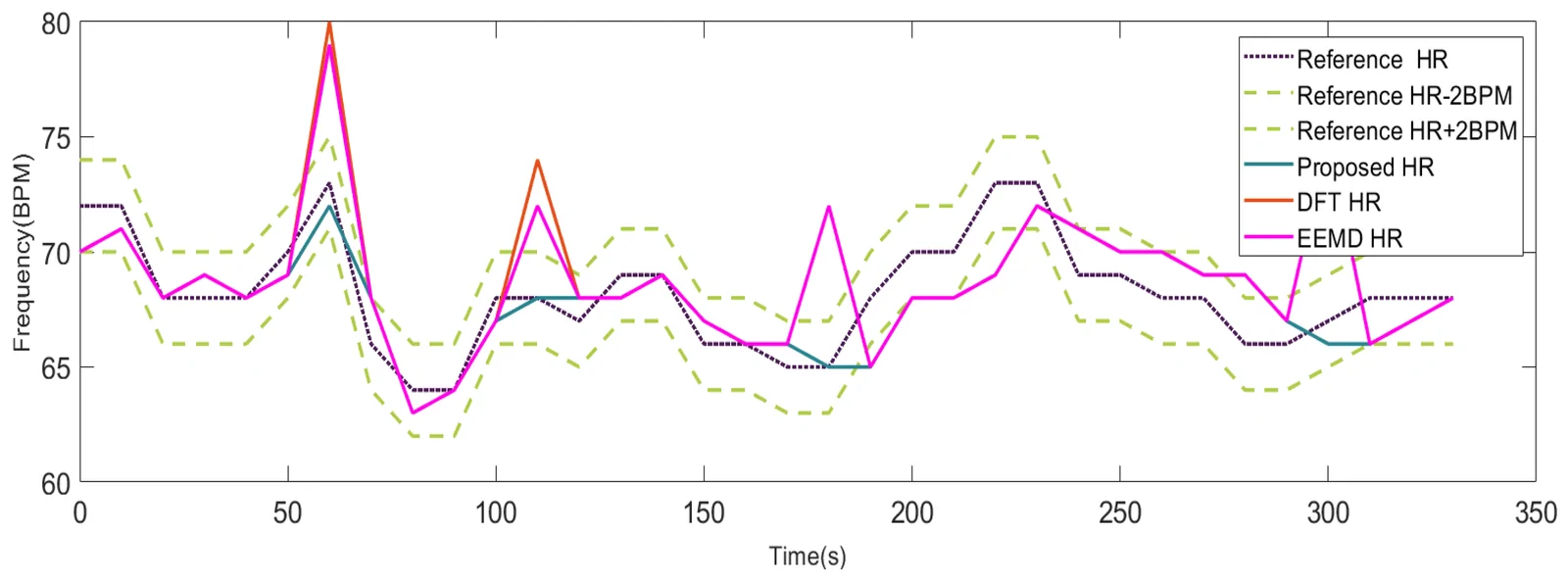
Long-term continuous heart rate estimation. Heart rates estimated using traditional DFT estimation, CEEMD algorithm estimation, and the proposed method, with reference to the actual values of the reference device, along with the corresponding 2 bpm error intervals.

To evaluate robustness, we processed a continuous 5 min recording using three methods: DFT only, EEMD, and the proposed combined framework (Figure 13). During intervals without significant respiratory harmonic interference, all three methods yielded comparable estimates. When strong respiratory harmonics were present, DFT-based estimates were frequently captured by harmonic peaks, producing large errors relative to the reference, while EEMD offered only partial improvement. The proposed method maintained stable tracking throughout the test period.

**Figure 14:**
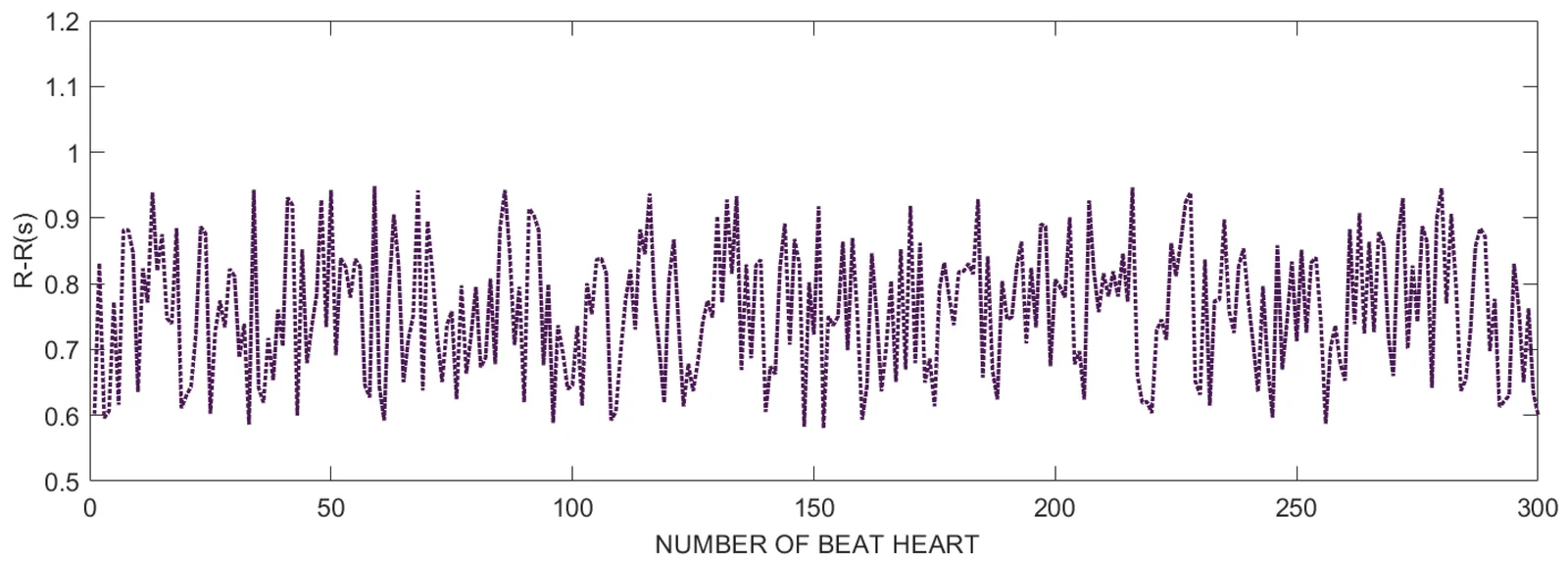
Tachogram of subject 1 (MALE1).

Figure 14 shows the tachogram of Subject 1, with heart rate estimates from the proposed method remaining stable between 50 and 80 bpm across the monitoring period, closely tracking the reference wearable sensor.

Quantitative evaluation across multiple subjects (Table 2) confirmed this pattern. In the absence of harmonic interference, all three methods achieved comparable accuracy. As monitoring duration increased and respiratory harmonics became significant, the accuracy of DFT and EEMD degraded substantially, while the proposed method preserved consistent performance. Under sustained harmonic interference, only the proposed framework maintained estimation reliability across the full test duration.

The SIR reported in Table 2 is defined as the ratio of the heartbeat spectral amplitude to the maximum respiratory harmonic spectral amplitude within the cardiac band (0.8–2.0 Hz) before processing. The SIR is calculated as

(22) $$SIR\;(\mathrm{dB})=10\;\cdot \;{log}_{10}(P\_heartbeat\;/\;max(P\_\;harmonic))$$

where P_heartbeat and P_resp_harmonic are the power spectral densities at the heartbeat fundamental frequency and the strongest respiratory harmonic within the cardiac band, respectively.

Average accuracy is defined as the percentage of heart rate estimates within each recording epoch for which the estimated heart rate satisfies |HR_est − HR_ref|/HR_ref ≤ 5%, where HR_ref is the heart rate measured by the reference wearable device (MI7).

To further evaluate the robustness of the proposed method, we conducted separate analyses on abnormal targets. For each recording interval, we individually screened and verified time segments where the estimation accuracy of the proposed method fell below 90%. Among all 30 recording intervals listed in Table 2, only one interval achieved an accuracy lower than 90%, and these problematic intervals corresponded to female 4 (46 years old) and female 5 (13 years old). The reduced accuracy stems from weakened cardiac displacement amplitude coupled with aggravated interference from respiratory harmonics. These abnormal samples reveal potential limitations when applying this method to subjects with weak mechanical coupling of the chest wall, and also point out directions for subsequent algorithm optimization.

As reported in Table 2, the proposed method exhibits consistent accuracy over all recording epochs with an SIR below 0 dB (meaning that, prior to processing, the respiratory harmonic interference overwhelms the heartbeat signal). This substantiates that our experimental dataset genuinely includes challenging harmonic-interference scenarios.

## 5. Discussion

The three-stage framework presented here enables radar-based heart rate estimation to remain accurate under conditions where respiratory harmonics coincide with the cardiac fundamental regime in which conventional DFT and EEMD methods systematically fail. The key interpretative insight is that the three stages are not merely additive but sequential enablers: coherent spatial aggregation raises the signal-to-noise ratio to a level where CEEMD-based time-frequency separation becomes effective, and the CEEMD stage in turn provides a sufficiently clean respiratory reference for the harmonic matching filter to resolve residual spectral overlap. Without the first stage, the CEEMD input would be dominated by noise; without the second, the harmonic filter would lack a reliable fundamental reference. This dependency chain explains why the combined framework succeeds where individual techniques applied in isolation do not.

The improvement over DFT-only and EEMD-only baselines is largest precisely where these methods are weakest: when a respiratory harmonic lies close to the cardiac fundamental and its amplitude exceeds or matches that of the heartbeat. In these regimes, DFT-based peak picking is captured by the harmonic rather than the cardiac peak, and EEMD alone cannot fully separate overlapping spectral components because its decomposition relies on frequency proximity rather than harmonic structure. The proposed framework’s ability to model the harmonic structure explicitly—rather than treating harmonics as noise—may explain its robustness across varied respiratory patterns and subject physiologies. Furthermore, the framework achieves this without the training data requirement that limits deep learning approaches [16,22] or the parameter sensitivity that constrains variational mode extraction methods [14], suggesting a practical advantage for deployment in unsupervised clinical or home monitoring scenarios.

The framework operates on standard commercial radar hardware. The gradient-descent-based harmonic matching is executed once per 10 s update window, and the CEEMD is terminated early when the IMF frequency falls below 0.2 Hz, which significantly reduces the computational load. At a 20 Hz sampling rate with a 10 s update interval, the per-window processing time on a typical embedded processor is estimated to be under 1 s, which is an order of magnitude shorter than the update interval, thereby leaving sufficient headroom for concurrent tasks. While a 10 s update interval is appropriate for steady-state monitoring of resting subjects, applications requiring faster response (<3 s) would benefit from reduced window lengths or more lightweight optimization solvers; we identify this as a direction for future work. Overall, the framework remains a practical candidate for continuous, unobtrusive vital sign monitoring in clinical and home healthcare settings where a 10 s update latency is clinically acceptable for heart rate trend tracking.

The proposed framework has several limitations that should be acknowledged. First, the harmonic matching and subtraction stage assumes a stationary breathing pattern within each 10 s window; transient breathing changes (e.g., sighs, breath-holds) can degrade the harmonic model accuracy. Second, the method has currently only been validated on a single-TX four-RX radar configuration with subjects seated at a fixed distance; performance at different distances, angles, and with MIMO configurations remains to be evaluated. Third, the CEEMD-based decomposition requires empirical selection of the number of ensemble realizations and noise amplitude, which may affect decomposition quality. Fourth, the framework has not been tested on subjects with irregular heart rhythms (e.g., atrial fibrillation) or respiratory conditions (e.g., tachypnea), where the fundamental assumptions of quasi-periodic vital sign signals may not hold. Finally, as noted above, validation under body movement conditions and with broader populations is needed before clinical deployment.

## 6. Conclusions

We have presented a signal processing framework that enables accurate radar-based heart rate estimation in the presence of respiratory harmonic interference without requiring training data, subject-specific calibration, or hardware modification. Experimental validation with a 77 GHz FMCW radar demonstrated that the proposed method maintains estimation accuracy within 5% of a reference wearable sensor under conditions where conventional DFT and EEMD methods exhibit substantial errors. The framework operates on standard commercial radar hardware with a computationally efficient pipeline suitable for real-time embedded deployment, making it a practical candidate for continuous, unobtrusive vital sign monitoring in clinical and home healthcare settings. The current implementation assumes a stationary seated subject, and future work should extend the approach to accommodate random body motion, multi-subject scenarios, and validation across broader patient populations in realistic clinical environments.

## Figures and Tables

**Table 1:** The magnitude and frequency of vital signs.

		Displacement	After Phase Unwrapping
	Frequency	Amplitude	Amplitude
Breath	0.2~0.8 Hz	1~12 mm	0.1~0.5 mm
heartbeat	0.8~2.0 Hz	0.1~0.5 mm	0.02~0.1 mm

**Table 2:** Average accuracy for different techniques, the reference device is MI7.

Subjects	BR Accuracy	Age	Minutes	SIR (dB)	DFT	EEMD	Proposed
male 1	100%		1	4.2	98%	98%	98%
	98%	26	3	2.1	96.7%	96.7%	97.5%
	100%		5	7.5	92.5%	93.4%	96%
male 2	100%		2	1.8	92%	92%	98%
	99%	29	6	2.9	94.7%	94.7%	97.5%
	98%		10	5.3	92.8%	94%	97%
	100%		1	6.1	92.5%	93%	96.4%
male 3	98%	22	3	4.7	94.5%	95%	97.3%
	97%		5	1.6	91.2%	92%	97.1%
	97%		2	4.7	96%	96%	96%
male 4	100%	53	6	4.3	95.2%	95.5%	95.5%
	99%		10	3.0	94.1%	94.5%	96%
	99%		2	5.8	97%	97%	97%
male 5	99%	12	6	3.5	95.3%	96%	97%
	100%		10	7.6	93.3%	94.2%	96%
female 1	100%		1	6.3	98%	98%	98%
	100%	23	3	5.9	92.7%	90.7%	98.2%
	99%		5	4.4	94.5%	92.3%	98.6%
female 2	98%		2	6.2	95%	95%	96%
	98%	25	6	2.9	93.7%	93.5%	96%
	95%		10	−3.1	89.5%	90.9%	95.3%
	100%		1	6.2	98.2%	98.2%	98.2%
female 3	100%	21	3	2.5	96.9%	96.9%	96.9%
	99%		5	7.3	94.5%	95%	97%
	99%		2	3.8	96.7%	96.7%	96.7%
female 4	97%	46	6	4.2	95.2%	95.2%	96.4%
	96%		10	3.4	89.5%	90.9%	95.3%
	99%		2	6.8	98%	98%	98%
female 5	93%	13	6	3.4	90%	90.3%	96.4%
	92%		10	−3.7	87.5%	87.5%	95.3%

## Data Availability

The raw data supporting the conclusions of this article will be made available by the authors on request.
